# Impact of hospital-acquired pneumonia on rehabilitation outcomes and mortality in patients after severe acquired brain injury: a retrospective cohort study

**DOI:** 10.3389/fneur.2026.1663431

**Published:** 2026-02-20

**Authors:** Valeria Pingue, Vittorio Gabba, Nicole Caggiano, Francesca Bottani, Gianluca Bellaviti, Antonio Nardone, Chiara Pavese

**Affiliations:** 1 Department of Clinical-Surgical, Diagnostic and Pediatric Sciences, University of Pavia, Pavia, Italy; 2 Istituti Clinici Scientifici Maugeri IRCCS, Neurorehabilitation and Spinal Units and Centro Studi Attività Motorie (CSAM) of Pavia Institute, Pavia, Italy

**Keywords:** disorder of consciousness, hospital-acquired pneumonia, rehabilitation, severe acquired brain injury, traumatic brain injury

## Abstract

**Background:**

Patients after severe acquired brain injury (sABI) have an increased susceptibility to systemic infections, which can severely compromise their rehabilitation process. In this study, we aimed to evaluate the impact of hospital-acquired pneumonia (HAP) on functional outcome and mortality in a large cohort of adult patients underwent inpatient rehabilitation from post-acute to 6 months after sABI.

**Methods:**

This observational retrospective cohort study included patients consecutively admitted after an acute sABI to Neurorehabilitation Unit for 6-month program between December 1, 2019, and December 31, 2022. Demographic data, etiology of sABI (traumatic vs. non-traumatic), length of stay, comorbidities, neurological impairment, presence of invasive devices (tracheostomy and nasogastric tube, percutaneous endoscopic gastrostomy), occurrence of HAP, and death during hospitalization were recorded. Functional assessments were evaluated at admission and discharge with the Functional Independence Measure (FIM), the Levels of Cognitive Functioning (LCF), and the Glasgow Outcome Scale-extended (GOS-E).

**Results:**

Of the 169 patients enrolled, 78 (46.2%) developed HAP during hospitalization. Of the 169 patients enrolled, 78 (46.2%) developed HAP during hospitalization. The multivariable logistic regression analysis identified as significant risk factors for the onset of HAP during hospitalization male sex (OR 0.313, *p* = 0.003), severity of brain injury (OR 0.825, *p* = 0.011), and the presence of nasogastric tube or percutaneous endoscopic gastrostomy due to the severity of dysphagia (OR 14.336, *p* = 0.024). The occurrence of HAP was the main predictor of poorer recovery at discharge in terms of FIM (*p* = 0.003) and LCF (*p* = 0.009), independent of other confounding variables at admission (e.g., age, severity, and etiology of brain injury). Furthermore, the occurrence of HAP was independently associated with a threefold (OR 3.088; *p* = 0.020) increase in mortality during inpatient rehabilitation.

**Conclusion:**

Severity of brain injury, male sex and presence of nasogastric tube or percutaneous endoscopic gastrostomy due to the severity of dysphagia may represent an early indicator of the risk of HAP in patients with sABI. Implementing preventive strategies and application of the care bundle to reduce the incidence of HAP, including improved oral hygiene, early mobilization, and appropriate management of dysphagia, may not only improve functional outcomes but also reduce mortality in patients undergoing rehabilitation after sABI.

## Introduction

Severe acquired brain injury (sABI) refers to brain damage occurring after birth due to traumatic, or non-traumatic causes (e.g., vascular, anoxic, infectious, metabolic, neoplastic) (1). The resulting severe impairment concerning sensory-motor and/or cognitive-behavioral functions (2) impacts patients and relatives’ lives, also making disability a social issue. Medical-rehabilitative intervention in the post-acute phase is essential to allow recovery of compromised abilities, with the ultimate goal of reinstating the maximum patient independence achievable. In this regard, important aspects to consider are clinical complications requiring elevated care assistance, which can hamper the recovery with a great impact on disability (3).

Patients with sABI are known to have an increased susceptibility to systemic infections, due to clinical complexity (4, 5) and immunosuppression, both induced by the severe primary brain injury (6–8). Moreover, prolonged hospitalizations, necessity of invasive devices (e.g., tracheostomy tube, central venous access, nasogastric tube or percutaneous endoscopic gastrostomy, bladder catheter), and dependence on health-care staff for daily care activities, result in an increased risk of contracting nosocomial infections or healthcare-associated infection (HAI), defined as infections arising 48 h after admission to a hospital facility (2). Recent studies (9–11) on the rehabilitation course after sABI showed that the occurrence of HAI in this intensive setting is related to poor functional outcomes, longer hospital length of stay (LOS), and higher mortality rate. Nosocomial pneumonia or hospital-acquired pneumonia (HAP), defined as bacterial lower respiratory tract infection that occurs 48 h or more after hospital admission (12), represents the most common infectious complications that occur in the neurointensive care unit (6, 10, 13). Indeed, hospitalized neurologic patients are especially prone to pneumonia due to common conditions such as a bedridden state, dysphagia, altered mental status, or respiratory muscle weakness (14). A cohort study on patients with mild to severe traumatic brain injury showed that HAP appears to be an independent negative prognostic factor for long-term functional recovery at 1, 2, and 5 years after injury (15). However, to date, a research gap exists in understanding the impact of HAP in the specific population of patients undergoing intensive rehabilitation programs after sABI (9). Therefore, the primary objective of this study was to analyze the impact of HAP on recovery and mortality in these patients, and the secondary objective was to identify possible variables associated with an increased risk of HAP.

## Materials and methods

### Study design and cohort definition

In this single-center retrospective cohort study, we included patients consecutively admitted to the Neurorehabilitation Unit of the ‘Istituti Clinici Scientifici Maugeri’ of Pavia, Italy, dedicated to the management and care of patients in the post-acute phase after sABI between December 1, 2019 and December 31, 2022. The inclusion criteria of the study were: (1) age ≥18 years; (2) admission to our rehabilitation unit within 3 months from sABI to continue clinical care and rehabilitation programs started at the Intensive Care Unit. We excluded patients with positive respiratory viral panel results in the absence of positive bacterial cultures or with mechanical ventilation. During the hospital stay, all patients underwent intensive multidisciplinary rehabilitation for up to 6 months consisting of an individual 90-min daily treatment program, 6 days a week.

The study design complied with the ethical guidelines of the Declaration of Helsinki, and was approved by the local Ethics Committee of Istituti Clinici Scientifici Maugeri (ICS Maugeri, ref. 2214 CE, June 19, 2018). All participants, or their authorized representatives, signed a written informed consent.

### Variables, data sources, and measurements

We collected from electronic clinical records the following data: age at injury, sex, LOS expressed in days of hospitalization, etiology of sABI, comorbidities (e.g., hypertension, diabetes mellitus, chronic renal failure, ischemic cardiopathy, hypothyroidism), neurological and functional assessments, presence of invasive devices (e.g., tracheostomy tube, nasogastric tube, percutaneous endoscopic gastrostomy), occurrence of HAP, death during hospitalization.

We defined HAP as any acute lower respiratory tract infection acquired after at least 48 h of admission to hospital (12), confirmed with positive bacterial microbiological culture and through chest radiography or computed tomography.

We used the Glasgow Coma Scale (GCS) to assess brain injury severity on admission. The GCS is a standardized system for evaluating the degree of neurological impairment and level of consciousness in all types of brain injury (16). The total score reflect the severity of brain damage, with scores of 3–8 indicating a severe injury, 9–12 a moderate injury, and 13–15 a mild injury (16).

Functional assessment was evaluated at admission and discharge with the Functional Independence Measure (FIM), the Levels of Cognitive Functioning (LCF), and the Glasgow Outcome Scale-extended (GOS-E). FIM is a standardized instrument designed to assess and monitor progress in functional status through 13 motor (FIM-m) and 5 cognitive items (FIM-c) in terms of independence (17). Each of the 18 items is graded on a scale of 1–7 based on the level of independence. The final total score ranges from 18 to 126, where 18 represents complete dependence/total assistance, and 126 represents complete independence (17). The LCF is a descriptive ordinal scale developed to monitor and classify deficit of responsiveness and degree of cognitive impairment in response to any stimulus (1, no response; 2, generalized; 3, localized; 4, confused–agitated; 5, confused-inappropriate; 6, confused–appropriate; 7, automatic–appropriate; 8 purposeful–appropriate) (18). Finally, GOS-E is an eight-point ordinal scale that broadly reflects the patient’s functional outcome with references to their dependence on others and community reintegration (1, death; 2, persistent vegetative state; 3, lower severe disability; 4, upper severe disability; 5, lower moderate disability; 6, upper moderate disability; 7, lower good recovery; 8, upper good recovery) (19).

### Statistical analysis

Data cleaning was performed before the analysis process, which did not reveal any missing or incorrect data. Categorical variables were expressed as absolute numbers and percentages, and differences were compared with the chi-square test, whilst non-normally distributed data were presented as the median and interquartile range (IQR), and differences were compared with the Mann–Whitney *U* test. Data were tested for normality of distribution via the Shapiro–Wilk test and log-transformed when needed, to correct for skewness. Multivariable logistic regression analysis was used to identify the potential risk factors of HAP and mortality during hospitalization. The Odds ratio (OR), 95% confidence interval (95% CI), and related significant values obtained from the regression analysis were reported.

A multivariable linear regression analysis was carried out to identify the variables’ potential predictive role on functional outcomes in terms of FIM, LCF, and GOS-E at discharge from the Neurorehabilitation Unit. The models included combinations of independent variables encompassing sex (M = 0, *F* = 1), age (<65 = 0, ≥65 = 1), severity and etiology (non-traumatic = 0, traumatic = 1) of sABI, LOS, presence of comorbidities, and occurrence of HAP. Moreover, each outcome measure was adjusted for its baseline value. Statistical analyses were performed using IBM SPSS Statistics version 21 (Somers, NY, USA). The threshold for statistical significance was set at *p* < 0.05.

## Results

### Clinical characteristics of the population at admission

**Figure 1:**
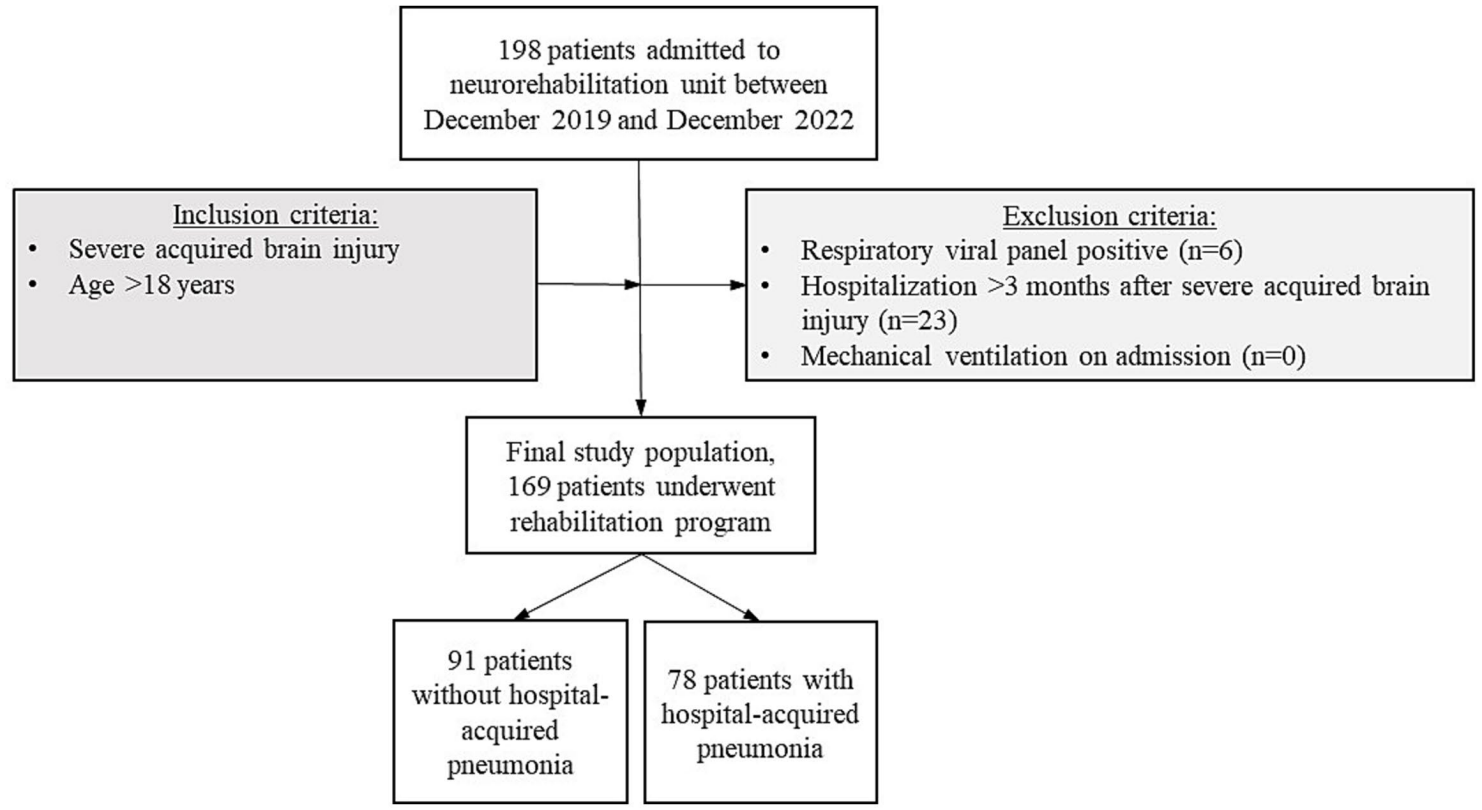
Participants’ enrolment and eligibility criteria flow chart.

The cohort included 169 patients (Figure 1). Among these, 78 (46.2%) developed HAP during hospitalization.

Demographic and clinical characteristics of patients included are presented in Table 1.

**Table 1:** Patients’ clinical-demographic characteristics at admission according to the occurrence or not of hospital-acquired pneumonia during inpatient rehabilitation.

Variables		All patients *n* = 169	Non-HAP *n* = 91 (53.8%)	HAP *n* = 78 (46.2%)	*p*
		*n* (%)	*n* (%)	*n* (%)	
Age	<65	105 (62.3)	58 (63.7)	47 (60.3)	0.750
	≥65	64 (37.9)	33 (36.3)	31 (39.7)	
Sex	Men	110 (65.1)	53 (58.2)	57 (73.1)	**0.052**
	Women	59 (34.9)	38 (41.8)	21 (26.9)	
Etiology	Traumatic	49 (29.0)	29 (31.9)	20 (25.6)	0.399
	Non-traumatic	120 (71.0)	62 (68.1)	58 (74.4)	
Length of stay		125.6(±77.6)	116.5(±76.6)	136.3(±77.8)	0.753
Comorbidities	Hypertension	80 (47.3)	40 (44.0)	40 (51.3)	0.358
	Diabetes mellitus	28 (16.6)	15 (16.5)	13 (16.7)	0.999
	Chronic renal failure	11 (6.5)	6 (6.6)	5 (6.4)	0.999
	Ischemic cardiopathy	38 (22.5)	15 (16.5)	23 (29.5)	0.064
	Hypothyroidism	10 (5.9)	7 (7.7)	3 (3.9)	0.344
Presence of device	Tracheostomy tube	127 (75.1)	58 (63.7)	69 (88.5)	**0.0003**
	Nasogastric tube	108 (63.9)	58 (63.7)	50 (64.1)	0.999
	PEG	40 (23.7)	13 (14.3)	27 (34.6)	**0.0034**

Data are expressed as mean ± SD or as absolute numbers and percentages. Comparisons between groups were performed using the *χ*^2^ test. Significant differences are reported in bold.

HAP, hospital-acquired pneumonia; PEG, percutaneous endoscopic gastrostomy.

The comparison of baseline characteristics shows no significant differences between the patients with and without HAP related to age, etiology (traumatic or non-traumatic), LOS, and presence of clinical comorbidities considered. On the contrary, a higher prevalence of HAP was observed in patients with tracheostomy tube (*p* = 0.0003) and percutaneous endoscopic gastrostomy (*p* = 0.0034) on admission.

**Figure 2:**
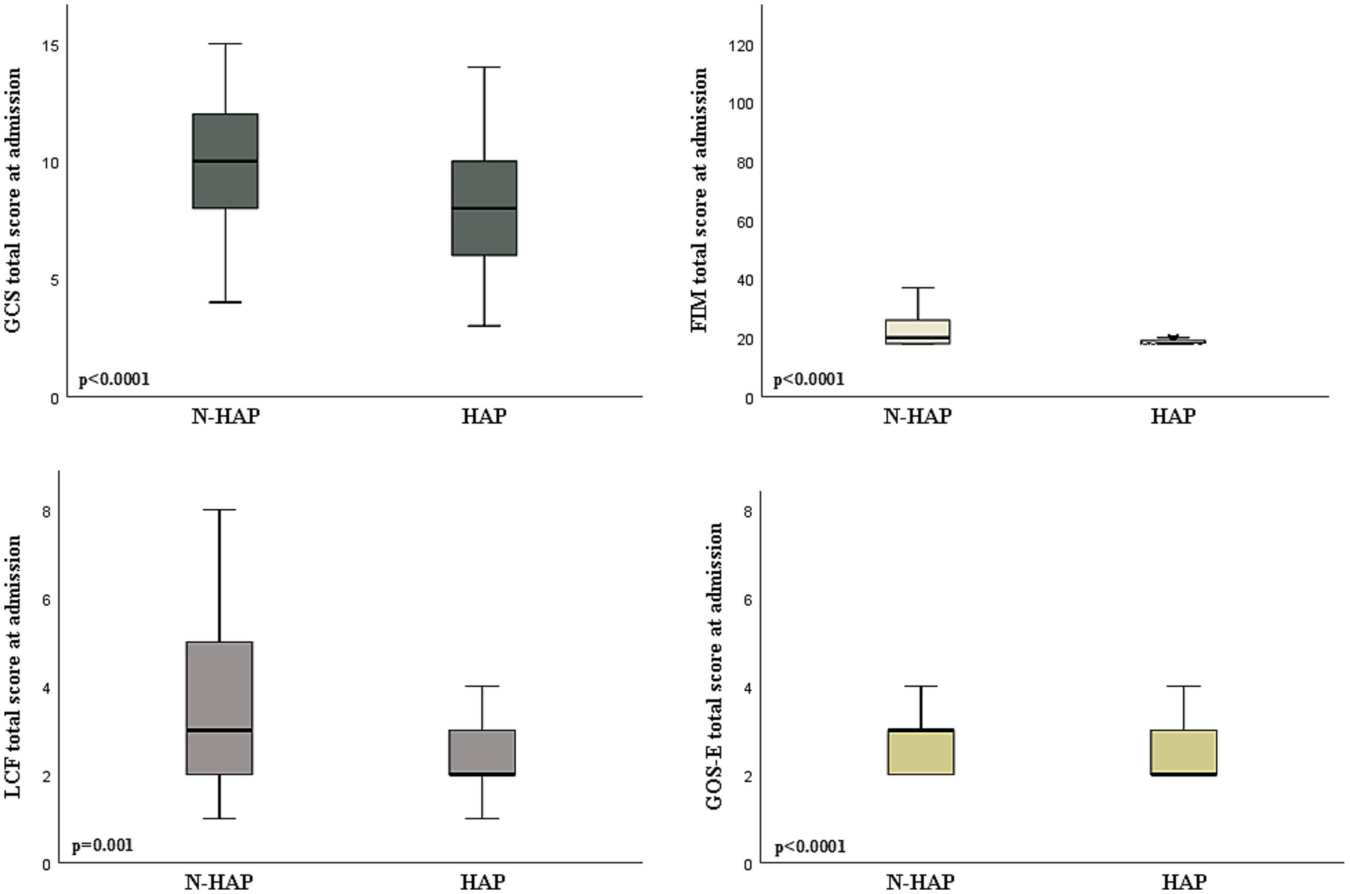
Comparison between patients according to the occurrence of hospital acquired pneumonia (HAP) or not (N-HAP) in terms of the neurological and functional assessment on admission to the rehabilitation unit. GCS, Glasgow Coma Scale; GOS-E, Glasgow Outcome Scale-extended score; FIM, Functional Independence Measure; LCS, Levels of Cognitive Functioning; DRS, Disability Rating Scale.

Figure 2 shows that patients who developed HAP during hospitalization presented a worse assessment on admission in terms of GCS (*p* < 0.0001), FIM (*p* < 0.0001), LCF (*p* = 0.001), and GOS-E (*p* < 0.0001).

The potential risk factors identified for the development of HAP are reported in Table 2.

**Table 2:** Multivariable logistic regressions showing potential risk factors for the onset of hospital-acquired pneumonia during inpatient rehabilitation in patients with severe acquired brain injury.

Covariates	HAP occurrence during inpatient rehabilitation after ABI (No = 0; Yes = 1)	
	OR (95% CI)	*p* value
Sex (M = 0; F = 1)	0.313 (0.146–0.672)	**0.003**
Age (<65 = 0; ≥65 = 1)	1.276 (0.590–2.762)	0.536
Glasgow Coma scale on admission	0.825 (0.711–0.958)	**0.011**
Etiology of injury (non-traumatic = 0; traumatic = 1)	0.520 (0.226–1.194)	0.123
Length of stay	0.999 (0.994–1.003)	0.556
Hypertension	0.970 (0.458–2.055)	0.937
Diabetes mellitus	1.405 (0.458–2.055)	0.506
Chronic renal failure	1.244 (0.288–5.373)	0.770
Ischemic cardiopathy	1.951 (0.812–4.686)	0.135
Hypothyroidism	0.290 (0.057–1.484)	0.137
Tracheostomy tube	1.598 (0.550–4.642)	0.389
Percutaneous endoscopic gastrostomy or nasogastric tube	14.336 (1.431–143.618)	**0.024**

HAP, hospital-acquired pneumonia; ABI, acquired brain injury; OR, odds ratio; CI, confidence interval. Significant risk factors are reported in bold.

The multivariable logistic analysis showed that male sex (OR 0.313, *p* = 0.003), severity of brain injury on admission (OR 0.825, *p* = 0.011), and presence of nasogastric tube or percutaneous endoscopic gastrostomy (OR 14.336, *p* = 0.024) were significantly associated with the development of HAP during inpatient rehabilitation, regardless of age, injury etiology, length of stay, and comorbidities.

### Functional outcome and mortality

Table 3 shows the possible predictors of recovery and functional outcome at discharge identified by the multivariable linear regression analysis.

**Table 3:** Multivariable linear regression analysis showing independent predictors of functional outcome at discharge (T1).

Independent variables	FIM T1 (*R*^2^ = 0.501)			LCF T1 (*R*^2^ = 0.459)			GOS-E T1 (*R*^2^ = 0.552)		
	*β*	SE (beta)	*p*	*β*	SE (beta)	*p*	*β*	SE (beta)	*p*
Sex (M = 0; F = 1)	−0.04	4.52	0.584	−0.07	0.334	0.300	0.04	0.19	0.518
Age (<65 = 0, ≥65 = 1)	−0.04	4.51	0.547	−0.03	0.337	0.699	−0.01	0.19	0.838
GCS at admission	0.28	0.89	**0.001**	0.31	0.076	**0.003**	0.51	0.04	0.091
FIM at admission	0.45	0.12	**<0.0001**	n.a.	n.a.	n.a.	n.a.	n.a.	n.a.
LCF at admission	n.a.	n.a.	n.a.	0.41	0.132	**<0.0001**	n.a.	n.a.	n.a.
GOS-E at admission	n.a.	n.a.	n.a.	n.a.	n.a.	n.a.	0.67	0.12	**<0.0001**
Etiology of injury (non-traumatic = 0; Traumatic = 1)	0.11	4.45	0.087	0.12	0.331	0.085	0.12	0.19	**0.050**
Length of stay	0.09	0.03	0.223	0.26	0.002	**0.001**	0.07	0.01	0.299
Hypertension	−0.02	4.20	0.708	0.04	0.310	0.541	−0.09	0.18	0.159
Diabetes mellitus	−0.03	5.74	0.664	0.06	0.421	0.359	0.04	0.24	0.521
Chronic renal failure	−0.16	8.13	**0.013**	−0.04	0.621	0.563	−0.03	0.36	0.561
Ischemic cardiopathy	−0.08	5.06	0.193	0.02	0.381	0.810	−0.05	0.22	0.419
Hypothyroidism	−0.01	9.05	0.845	0.01	0.663	0.842	0.06	0.38	0.277
HAP	−0.20	4.47	**0.003**	−0.19	0.335	**0.009**	−0.07	0.19	0.289

FIM, Functional Independence Measure; LCF, Levels of Cognitive Functioning; GOS-E, Glasgow Outcome Scale-extended; HAP, hospital-acquired pneumonia; GCS, Glasgow Coma Scale; n.a., not applicable.

Significant predictors are reported in bold.

Occurrence of HAP was a main predictor of worse recovery at discharge in terms of FIM (*p* = 0.003) and LCF (*p* = 0.009). Other relevant predictors of poor functional outcome included older age, severity of neurological impairment on admission, non-traumatic etiology, chronic renal failure, and LOS.

Death during inpatient rehabilitation was documented in 36 (21.3%) patients after sABI, with a higher prevalence (*p* = 0.0006) in subjects that developed HAP (25 patients) compared to the other group (11 patients) (not shown). Table 4 reports the potential risk factors for mortality identified during hospitalization among the variables considered in our cohort.

**Table 4:** Multivariable logistic regressions showing potential risk factors for mortality during inpatient rehabilitation after severe acquired brain injury.

Covariates	Death during inpatient rehabilitation (No = 0; Yes = 1)	
	OR (95% CI)	*p* value
Sex (F = 1; M = 0)	1.213 (0.481–3.063)	0.682
Age (<65 = 0; ≥65 = 1)	4.493 (1.785–11.307)	**0.001**
Glasgow coma scale on admission	0.837 (0.714–0.982)	**0.029**
Etiology of injury (non-traumatic = 0; traumatic = 1)	0.260 (0.075–0.903)	**0.034**
Hypertension	1.464 (0.592–3.622)	0.409
Diabetes mellitus	1.070 (0.348–3.290)	0.906
Chronic renal failure	1.569 (0.317–7.777)	0.581
Ischemic cardiopathy	1.082 (0.409–2.866)	0.874
Hypothyroidism	2.209 (0.417–11.706)	0.352
Hospital acquired pneumonia	3.088 (1.190–8.012)	**0.020**

Significant risk factors are reported in bold.

Occurrence of HAP was independently associated with a threefold increase in mortality (OR 3.088; *p* = 0.020) during inpatient rehabilitation. Other risk factors of mortality were older age at diagnosis (OR 4.493; *p* = 0.001), non-traumatic etiology (OR 0.260; *p* = 0.034), and severity of brain injury (OR 0.834; *p* = 0.029) on admission.

## Discussion

In the present study, we aimed to evaluate whether the occurrence of HAP could impact functional outcome and mortality in post-sABI rehabilitation patients, and to identify possible risk factors for its occurrence. Our main findings indicate that the occurrence of HAP in the post-acute phase after sABI emerged as an independent predictor of poor recovery at discharge in terms of FIM, LCF, and GOS-E, and a risk factor of mortality during inpatient rehabilitation, as well as older age, severity, and non-traumatic etiology of brain injury.

Most of the available data on HAP are focused on patients in intensive and acute care units (15). A recent prospective study on moderate-to-severe TBI patients showed that individuals with HAP during acute hospitalization have worse long-term prognosis and greater hospital resource utilization (16). Compared to this study (16), we recorded a higher incidence rate (46.2%) of HAP during inpatient rehabilitation. Several factors were associated with an increased risk of HAP in our cohort; in particular, more than half of the patients presented with medical devices due to the severity of neurological impairment. Indeed, in our study, as previous reported (9, 16, 20), patients who developed HAP during hospitalization had mostly a tracheostomy tube and percutaneous endoscopic gastrostomy at the time of admission to our Unit. The presence of a nasogastric tube or percutaneous endoscopic gastrostomy correlated with the severity of dysphagia and represented a statistically significant risk of HAP. As expected, lower GCS on admission was another significant predictor of HAP, confirming that severe neurological damage predisposes patients to pulmonary infections through mechanisms such as impaired swallowing reflex, altered consciousness, and secondary immunodeficiency (9). Although patients with sABI are at greater risk for developing both HAP and poor outcomes, multivariable regression analysis, after adjusting for potential confounders including age, sex, severity, and etiology of ABI, confirmed the harmful effects of HAP on functional assessment at discharge in terms of changes in FIM, LCF, and GOS-E from admission.

Nursing interventions play a significant role in the management of highly dependent patients due to severe neurological impairment. Recently, studies have evidenced that the application of the care bundle (feeding and body position management, oral care, and respiratory tract management) is associated with a lower incidence of stroke-associated pneumonia (21, 22).

The pathogenesis of HAP remains complex due to immunosuppression induced by the severity of brain damage, dysphagia, and altered cough reflex (23). However, previous reviews have indicated that interventions that target potential modifiable risk factors may reduce HAP in acute settings, including improved oral hygiene, hand hygiene, early mobilization, identification and appropriate management of dysphagia, particularly in post-stroke patients (24).

Pulmonary rehabilitation is challenging in patients after an sABI due to impaired consciousness. Nonetheless, preventive respiratory strategies for managing airway clearance should be an integral part of the rehabilitation program after a sABI, given the relevant impact on outcome and mortality of HAP.

### Study limitations

This study has some limitations. First, the retrospective design implies a review of clinical files not originally aimed at collecting data for research, with a risk of selection and recall biases, and possible missing information. In particular, the severity of dysphagia is only reported through indirect indicators (presence of nasogastric tube or percutaneous endoscopic gastrostomy). However, the study sample size was very large, and the well-characterized cohort of patients was hospitalized in a tertiary referral center. Additionally, we included variables such as age, sex, comorbidities, and invasive devices, which may influence the risk of HAP and rehabilitation outcomes. However, we reduced this bias by weighing the multivariable analysis for the severity of the neurological damage. Finally, the retrospective nature of the study did not allow us to clarify the causal relationship between HAP and poor outcome after sABI. Further studies are needed to investigate the mechanisms underlying these associations.

### Conclusions and clinical implications

In summary, our findings highlight HAP as a potential risk factor affecting recovery and mortality in patients with sABI. These results underline the importance of careful monitoring and preventive strategies to reduce the incidence of HAP in patients undergoing rehabilitation after sABI. Implementing a targeted rehabilitation strategy, in particular in patients with medical devices and dysphagia due to severe neurological status on admission, may not only improve functional outcomes but also reduce mortality.

## Data Availability

The raw data supporting the conclusions of this article will be made available by the authors, without undue reservation.
